# What is isostructurality? Questions on the definition

**DOI:** 10.1107/S2052252523010436

**Published:** 2024-01-01

**Authors:** Petra Bombicz

**Affiliations:** a Centre for Structural Science, HUN-REN Research Centre for Natural Sciences, Magyar Tudósok körútja 2, Budapest 1117, Hungary; University of Iowa, USA

**Keywords:** isostructurality, symmetry, extent of similarity, supramolecular interaction

## Abstract

Investigation of isostructurality leads to a deeper understanding of close-packing principles and contributes to the ability of crystal engineering. A given packing motif may tolerate small molecular changes within a limit. Slight alterations of a crystal packing arrangement are carried out in order to fine-tune the structural and macroscopic properties, keeping the balance of the spatial requirements and electrostatic effects of the altered molecules in the crystals, preserving their isostructurality. Even so, the definition of isostructurality is not explicit about several issues. Are the corresponding structures required to have the same stoichiometry, *Z*′, symmetry elements and the same space group? Because it is not obvious in the definition, studies on structure analysis and software calculating various numerical descriptors developed for the quantitative comparison of the degree of similarity of isostructural crystals self-define their criteria. The extent of the difference between corresponding crystal structures referred to as isostructural is not limited. Should it be determined numerically? There is nothing in the definition about a demand for similar supramolecular arrangements in isostructural crystals. Should the similarity of supramolecular interactions be a criterion of isostructurality? The definition of isostructurality deserves reconsideration regarding symmetry, measure of similarity and formation of supramolecular interactions.

## Abstract 2

The phenomenon and definition of isostructurality are discussed. It deserves reconsideration regarding the aspects of symmetry, measure of similarity and formation of supramolecular interactions.

## 1. Introduction

An increasing number of new substances with tailor-made properties are produced by crystal engineering (Desiraju, 2018 ▸, 2001 ▸, 2002 ▸; Russell & Ward, 1996 ▸). The attempt to fine-tune structural properties requires mastering the supramolecular packing architecture (Galcera *et al.*, 2013 ▸; Bombicz *et al.*, 2014 ▸; de Vries *et al.*, 2016 ▸; Bombicz, 2017 ▸). A series of crystal structures with a gradual transition that conserves the isostructurality of the crystals can be prepared by introducing fine chemical changes. The arrangements of molecules in crystals can be firmly influenced by the application of substituents, by changing their placement and/or chemical composition, or by using compounds with a similar chemical composition in multi-component structures. The balance of spatial requirements and electrostatic effects ultimately determines the molecular arrangement. In isostructural crystals, both the placement of the molecules and the conformation of flexible molecules may adjust to the chemical and supramolecular features. The formation of isostructural crystals is a delicate energy balance between the energy loss caused by the expansion of the framework and the addition of intermolecular interactions (Jia *et al.*, 2023 ▸). A given packing motif may tolerate small molecular changes, and the structures remain isostructural within a limit despite chemical changes. Isostructural crystals are highly similar in their packing arrangement, but may differ to a lesser or greater extent in physico-chemical properties (Dey *et al.*, 2016 ▸; Wood *et al.*, 2012 ▸), like seeding and crystal growth, recognition processes, stability, and biological activity. The various forms of packing similarity between organic crystals (isotypic, homeotypic, isomorphic, isometric, homeostructural *etc*.) were summarized by Kálmán *et al.* (1993 ▸).

The investigation of isostructurality leads to a deeper understanding of the close-packing principles, the role of molecular conformation, supramolecular interactions and symmetries in order to be able to perform the directed manipulation of the molecular packing arrangements (Wood *et al.*, 2012 ▸; Fábián & Kálmán, 2004 ▸). Although, in cases of occurrence, isostructurality is often mentioned as a qualitative delineation in structural studies, its quantitative description has also been developed. Notwithstanding, the recognition of isostructurality is not necessarily straightforward.

## 2. Discussion

Isostructurality calculations and statistical analyses are efficient tools for the discovery of isostructural crystals (Bombicz *et al.*, 2020 ▸). The calculation of the cell similarity (π) and isostructurality (*I*_s_) indices (Kálmán *et al.*, 1993 ▸, 1996 ▸; Kálmán & Párkányi, 1997 ▸), as well as molecular isometricity indices (Kálmán *et al.*, 1993 ▸; Macrae *et al.*, 2008 ▸) completed with a prior multivariate data analysis of the structural data, contribute to the facile recognition and numerical characterization of isostructural crystals. The cluster analysis is a quick and easy-to-use tool to discover isostructurality before performing a thorough structure analysis to filter isostructural crystals out from the abundance of structures with similar cell parameters or even similar internal arrangements. The multivariate data analysis is based only on mathematical data without any prior knowledge of substitutions, space groups and structural analysis. The numerical descriptors of the isostructurality make the quantitative comparison of the degree of similarity possible. There are a few software packages developed and made available for the crystallographic community to calculate isostructurality. The software *ISOS* performs cell similarity (π) and isostructurality (*I*_s_) indices calculations. The isostructurality index is calculated by fitting the crystal coordinates of the identical non-hydrogen atoms within the chosen asymmetric unit, which is the closest to the origin in the related structures (Bombicz *et al.*, 2020 ▸; Kálmán *et al.*, 1993 ▸; Kálmán, 1996 ▸; Kálmán & Párkányi, 1997 ▸). The volumetric measure of isostructurality (*I*_v_) is defined as the percentage ratio of the overlapping volume of molecules in the analyzed structures to the average of the corresponding molecular volumes, calculated in the unit cell (Fábián & Kálmán, 1999 ▸). It is capable of treating disordered structures. The *XPac* dissimilarity index (*x*) (Gelbrich *et al.*, 2012 ▸) employs a particular strategy whereby corresponding internal coordinates, particularly intermolecular angular parameters of the concerning crystal structures, are compared. This makes it possible to identify crystal packing fragments of a similar geometry: supramolecular constructs (SCs). A given molecular crystal structure is represented by a cluster comprising a general molecule and the *n* (typically *n* = 14) molecules which form its first environment. The *Mercury Crystal Packing Similarity* tool from *CSD-Materials* (Macrae *et al.*, 2008 ▸) compares structures and quantifies (RMSD) the degree to which these are similar by identifying how many molecules within a reference cluster (typically *n* = 20) match the associate cluster of the comparison structure by overlaying them. The more molecules that overlay within the set geometric tolerances (distance and angle), the more similar the packing is between the structures. These classifications allow variation in symmetry.

Powder diffractograms are unique fingerprints: PXRD is suitable for the identification of individual crystals within an isostructural series. Nonetheless, a match between PXRD patterns of isomorphic crystals can be perceived on occasion (Wood *et al.*, 2012 ▸; Ranjan *et al.*, 2020 ▸).

**Figure 1:**
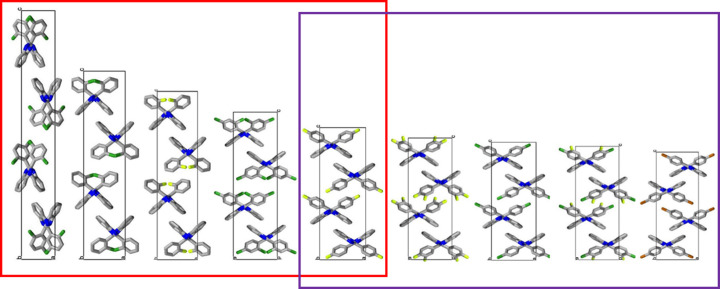
Unit cells of nine members of the exemplary isostructural series of halogen-substituted PBI derivatives, all in the *Pbca* space group (Bombicz *et al.*, 2020 ▸). The crystals in the series show gradual alteration. The determining supramolecular interaction is *X*⋯π in the structures highlighted by the red frame, whereas the *X*⋯*X* intermolecular interaction is decisive in the structures within the purple frame. The structure within the overlap contains both types of intermolecular interactions, thus it belongs to both subgroups, and it acts like a switch within the series.

For an exemplary isostructural series of halogen-substituted 2-phenyl­benzimidazole (PBI) derivatives, how the isostructural similarities of the *Pbca* crystals can be described by numerical descriptors and how the relationship between the molecular and supramolecular properties and the structural features can be revealed and characterized were presented (Bombicz *et al.* 2020 ▸). The multivariate data analysis, as well as the cell similarity, isostructurality and molecular isometricity calculations were applied to identify and quantify the structural similarities of the PBI compounds. The crystal structures in the series of the ten isostructural crystals show gradual alteration, they look like a lab jack lifted to different heights (Fig. 1 ▸). The packing arrangements of the neighboring structures in the fine-tuned series of the molecular sequence are highly similar. However, the length of the ‘*a*’ unit cell axis is increased by 67% and the length of the ‘*c*’ unit cell axis is decreased by 43% within the series when comparing the first and the last members. The structures of the two extremes, from the two ends of the series, have low similarity. How far can we say that two structures from the series are isostructural: is the *n*th member isostructural with the (*n* + 1)th, with the (*n* + 2)th, (*n* + 3)th,…, (*n* + 10)th? On the other hand, the preference of the determining intermolecular interactions switches in the middle of the molecular sequence in the isostructural PBI crystal series investigated. The difference in the supramolecular interactions divides the structures into two subgroups, while the similar molecular arrangement in the structures is maintained in the *Pbca* space group. The adjustment of the motif of the intermolecular interactions in the series could be revealed through supramolecular investigations, it is not indicated by the numerical descriptors of isostructurality. The limit of the tolerance of the given molecular arrangement was challenged, the isostructurality terminates with certain chemical modification and the space group transforms from *Pbca* into *P*2_1_/*c*. The change in space group indicates the loss of a twofold screw axis symmetry, as *P*2_1_/*c* is a maximal non-isomorphic subgroup of *Pbca*.

The prerequisites of isostructurality are similar composition and conformation of the compounds, with analogous molecular arrangement in the crystals. The examples investigated open up three questions on isostructurality:

(1) Are the corresponding structures required to have the same stoichiometry, space group, *Z*′ and the same symmetry elements? Studies on structure analysis and software that calculate isostructurality self-define these aspects, showing no agreement on these issues.

(2) How large can the extent of difference be between the corresponding crystal structures that we may still consider them as being isostructural? Should this be determined numerically?

(3) Isostructurality investigations need to be completed with a similarity check of the supramolecular interactions. There are crystals whose cell parameters are similar, space groups are the same and arrangements of the molecules are analogous, the only difference is in the preference of the intermolecular interactions. Can we consider them to be isostructural?

According to the IUCr definition (https://dictionary.iucr.org/Isostructural_crystals):

Two crystals are said to be isostructural if they have the same structure, but not necessarily the same cell dimensions nor the same chemical composition, and with a ‘comparable’ variability in the atomic coordinates to that of the cell dimensions and chemical composition.

There are questions in the definition of isostructurality: what do the ‘same structure’, the ‘same cell dimensions’, the ‘same chemical composition’ and ‘comparable variability’ of atomic coordinates mean? Isostructural crystals have ‘different chemical compositions’, highly similar ‘structural arrangements of molecules’ and ‘differences in the cell dimensions’, and the ‘variability in the atomic coordinates’ can be explicitly described numerically (Table 1 ▸).

## 3. Conclusions

The definition of isostructurality and the different calculations of the isostructurality index take into account both the differences in the conformation of the molecule(s) and their positional differences at the same time. It is a question of what the extent of differences is the limit to consider the structures as isostructural. The expression in the present definition of ‘same structure’ is not explicit about the symmetry restrictions, whether it requires the compared structures to have the same symmetry elements. In the case of a wide interpretation, the isostructural crystals with the same space group represent a subgroup, which should be uniquely identified, because of its significance. The expression ‘same structure’ in the present definition is not explicit about the supramolecular interactions, whether it requires the structures compared to have identical intermolecular systems. In the case of a wide interpretation, the isostructural crystals with the same supramolecular arrangement represent a subgroup, which also deserves distinguishing, since the supramolecular similarity is a condition of the similar properties of the crystals. If narrow interpretations are preferred, the definition of isostructurality needs to be more specific.

Two crystals may be said to be isostructural if they have similar chemical compositions, the arrangement of the molecules in their crystals are highly similar (subject to symmetry aspects, molecular conformation and molecular placement), their cell dimensions and variability in atomic coordinates do not exceed a given extent, and the systems of the supramolecular interactions match. To fit the state-of-the-art crystallography based on the delineated arguments above, the definition of isostructurality needs reconsideration regarding the aspects of symmetry, measure of similarity and formation of supramolecular interactions.

## Figures and Tables

**Table 1:** Aspects of isostructurality detailed in the IUCr definition and this paper

Criteria	IUCr definition	Present paper
Chemical composition	Not necessarily the same	Similar
Cell dimensions	Not necessarily the same	Do not exceed a given extent
Structure, *e.g.* molecular arrangement in the crystal	Same	Highly similar (subject of symmetry aspects, molecular conformation and molecular placement)
Atomic coordinates	Comparable variability	Do not exceed a given extent
Supramolecular interactions	(Not mentioned)	Match
